# Optimisation of Nitrogen, Phosphorus, and Potassium for Soilless Production of *Cannabis sativa* in the Flowering Stage Using Response Surface Analysis

**DOI:** 10.3389/fpls.2021.764103

**Published:** 2021-11-17

**Authors:** Lewys Bevan, Max Jones, Youbin Zheng

**Affiliations:** ^1^School of Environmental Sciences, University of Guelph, Guelph, ON, Canada; ^2^Department of Plant Agriculture, University of Guelph, Guelph, ON, Canada

**Keywords:** cannabis, cannabinoids, nutrient, nitrogen, phosphorus, potassium

## Abstract

Following legalisation, cannabis has quickly become an important horticultural crop in Canada and increasingly so in other parts of the world. However, due to previous legal restrictions on cannabis research there are limited scientific data on the relationship between nitrogen (N), phosphorus (P), and potassium (K) supply (collectively: NPK) and the crop yield and quality. This study examined the response of a high delta-9-tetrahydrocannabinol (THC) *Cannabis sativa* cultivar grown in deep-water culture with different nutrient solution treatments varying in their concentrations (mg L^–1^) of N (70, 120, 180, 250, 290), P (20, 40, 60, 80, 100), and K (60, 120, 200, 280, 340) according to a central composite design. Results demonstrated that inflorescence yield responded quadratically to N and P, with the optimal concentrations predicted to be 194 and 59 mg L^–1^, respectively. Inflorescence yield did not respond to K in the tested range. These results can provide guidance to cultivators when formulating nutrient solutions for soilless cannabis production and demonstrates the utility of surface response design for efficient multi-nutrient optimisation.

## Introduction

Drug-type cannabis (*Cannabis sativa*) is an important horticultural crop grown for medicinal and recreational purposes. Historically, many countries have prohibited the cultivation of drug-type cannabis which consequently provided a significant barrier to research on this crop. However, change in social attitudes towards consumption of cannabis has led to the repeal of cannabis prohibition in several countries/regions around the world. Following the 2018 repeal of cannabis prohibition in Canada, production of cannabis has quickly become an important part of the Canadian horticulture industry worth billions of dollars annually (Zheng, 2021). However, cannabis cultivators still lack scientific information about optimal growing conditions, such as supply of mineral nutrients, to help maximise crop yields, quality, and profits while minimising environmental impacts.

Proper supply of mineral nutrients is essential for efficient and sustainable cultivation of any crop. Among the most important nutrients for plants are nitrogen (N), phosphorus (P), and potassium (K). However, few studies have investigated the response of cannabis to these nutrients. As a result, cannabis cultivators often rely on nutrient recipes developed by fertiliser companies, or by community consensus based on previously clandestine production. This poses a problem because deficient or excessive supply of nutrients may reduce yield (Caplan et al., 2017a,b) or lead to environmental pollution from runoff of excess nutrients (Beerling et al., 2014; Zheng, 2018). Nutrient runoff is an issue in many agricultural areas of the world because excess nutrients, specifically P, can lead to the eutrophication of water bodies (Schindler et al., 2016). In Ontario (the Canadian province in which this study was conducted) disposal of waste greenhouse nutrient solution, including from cannabis production facilities, is regulated by law at considerable cost to the cultivators (Ontario Ministry of Agriculture Food and Rural Affairs, 2019). An understanding of cannabis’ mineral nutrient requirements can help us better synchronise nutrient supply and demand to maximise production while reducing nutrient waste and resulting environmental impacts.

Recent peer-reviewed studies have started to examine the response of *Cannabis* to mineral nutrients, but this area of research remains largely unexplored. These studies indicate the optimal N supply for both vegetative and flowering stages of cannabis production using conventional fertilisers is approximately 160 mg L^–1^ (Saloner and Bernstein, 2021). Plants supplied with N below 160 mg L^–1^ during the vegetative stage saw reduced photosynthetic capacity and plant growth, and during the flowering stage saw reduced inflorescence yield, though cannabinoid concentrations (not total production) were greater at extremely low N rates. The optimal N supply for plants grown with liquid organic fertilisers seems to be higher, with the highest yields being achieved with an organic N supply of approximately 390 and 260 mg L^–1^ for the vegetative and flowering stages, respectively (Caplan et al., 2017a,b). Given the limited number of studies and the relative importance of N on plant growth and development, collecting more information about cannabis response to N are needed to establish more accurate recommendations.

Phosphorus nutrition has long been a focus in cannabis cultivation. Growers often supply plants with relatively high P concentrations (up to 200 mg L^–1^) during the flowering stage based on a belief that high P promotes flower development. However, there is little evidence to support this practice. A recent study found that cannabis plants in the vegetative stage supplied with 100 mg L^–1^ P performed similar to those supplied with 30 mg L^–1^ P (Shiponi and Bernstein, 2021). High P concentration in the nutrient solution creates a situation where environmental pollution from excess P is more likely. Clearly, the practice of supplying cannabis with high concentrations of P needs to be evaluated.

While there are no published studies examining the effect of K on inflorescence quality, some recent studies have looked at how K impacts inflorescence yield. Yield of aquaponically grown cannabis (g/plant) increased linearly with increasing nutrient solution K concentration in the range of 15–150 mg L^–1^ (Yep and Zheng, 2020). The nitrogen concentration (75 mg L^–1^) used by Yep and Zheng (2020) reflects that of a typical aquaponic solution, but this N concentration is fairly low compared to conventional hydroponic nutrient solutions and may have been a limiting factor for plant growth and yield (Yep et al., 2020b). For the vegetative stage, cannabis plants supplied with 15 mg L^–1^K had reduced growth and displayed foliar symptoms characteristic of K deficiency, while plants that received 60–240 mg L^–1^ K produced substantially more biomass and did not display K deficiency symptoms (Saloner et al., 2019). Although there is a lack of recommendations based on scientific research, some fertiliser companies are recommending 300–400 mg L^–1^K. More research is needed to determine the optimal nutrient solution K concentration during cannabis flowering in soilless production systems when other nutrient elements are not limiting.

A challenge in developing fertiliser recommendations is that the number of combinations of nutrient concentrations that can be empirically tested is limited due to logistical and statistical considerations. As a result, most nutrient studies have a limited range of nutrient compositions that can overlook potential nutrient interactions across a broad range of nutrient compositions. Studies on cannabis response to nutrients so far have either investigated different concentrations of one nutrient while holding the others constant (Saloner et al., 2019; Saloner and Bernstein, 2020, 2021; Shiponi and Bernstein, 2021), or provided different concentrations of NPK in a set ratio (Caplan et al., 2017a,b; Bernstein et al., 2019). Neither of these approaches can evaluate nutrient interactions, which could have substantial impacts on the recommendations of optimum application rates.

Response surface methodology (RSM) is an alternative experimental design capable of concurrently optimising multiple factors over a wide range of levels using fewer experimental units compared to traditional designs (Myers et al., 2016). The efficiency of this design is achieved by using fewer experimental units which conserves space, time, and resources. Nutrient solution optimisation has been approached by some researchers as a “mixture system” which is a type of multifactor optimisation similar to response surface analysis (De Rijck and Schrevens, 1998, 1999a). However, the experimental design of a mixture system only optimises the nutrient composition of the solution but not the overall nutrient concentration as the design maintains a constant total nutrient supply in the solution. RSM allows the optimisation of both the nutrient solution composition and the concentrations of individual components without this limitation. Given the high cost of cannabis and growing space being limited to government-approved production facilities, the reduced number of experimental units required for a RSM approach is an advantage over conventional experimental designs.

The objective of this study was to determine the optimal concentrations of NPK for the flowering stage of cannabis in a soilless production system using the RSM approach.

## Materials and Methods

### Plant Material and Growing Conditions

**FIGURE 1:**
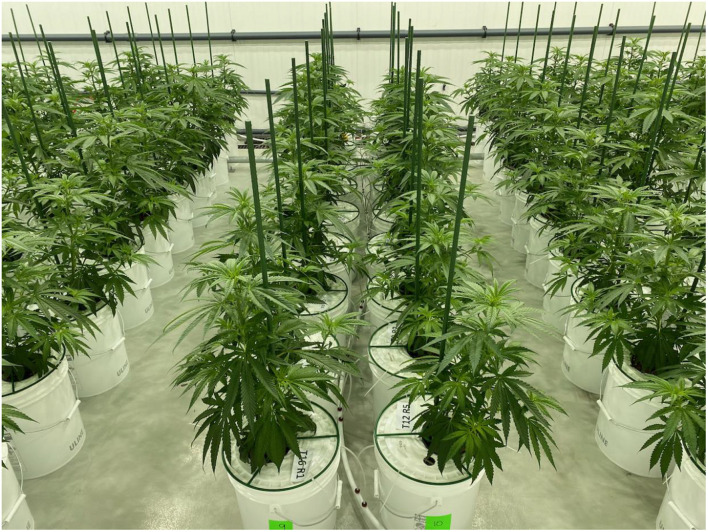
Rows of deep-water culture units containing trial plants at the end of the 3-week vegetative stage.

The experiment was conducted in a controlled-environment growth room at a Health Canada approved cannabis production facility located in Southern Ontario. A clonal selection of a high delta-9-tetrahydrocannabinol (THC), low cannabidiol (CBD) *C. sativa* cultivar “Gelato” was used for this trial. Plants were grown in deep-water culture (DWC) systems. Each DWC unit used a 19 L white plastic bucket (36 cm height × 30.5 cm top outside diameter × 26.4 cm bottom outside diameter) as the nutrient solution reservoir. DWC units were placed on the floor in five double rows of ten DWCs each (i.e., 100 DWCs total), each with one plant, spaced ten cm between adjacent units, 15 cm within the rows, and a one metre aisle-space between rows (Figure 1). Uniform 2-week-old cuttings (∼15 cm tall, 5–6 nodes trimmed to 3–4 leaves) rooted in rockwool cubes were transplanted into each DWC unit using a mesh pot (FHD Plastics, 0.62 L, 10.3 cm height × 12.5 cm diameter) filled with 8–16 mm expanded clay pebbles (Liapor, Hallerndorf, Germany) and inserted flush to the top of the bucket lids, with the bottom three cm of the mesh pot submerged in the nutrient solution. Each DWC bucket was supplied with nutrient solution and had an air-stone (Pawfly ASC030, 30 mm height × 18 mm diameter) providing 1.5 litres of air per minute to continuously mix and aerate the solution. The nutrient solutions in all DWC units were drained and replaced with 17 L of fresh nutrient solution weekly.

Plants were grown in the DWC systems vegetatively, under 18/6-h light/dark conditions, for 3 weeks before switching to a 12/12-h light/dark (i.e., short-day) photoperiod, to induce flowering. Plants were grown under short-day conditions for 7 weeks before being harvested. Light was provided by 1000 W metal halide bulbs at an average canopy-level photosynthetically photon flux density of 570 μmol m^–2^ s^–1^. The air temperature and relative humidity were set at 25°C and 65%, respectively. There was no CO_2_ supplementation.

### Experimental Design and Treatments

A three-factor (i.e., N, P, and K), second order central rotatable composite design was used to model cannabis responses to these mineral nutrients. Following a response surface design (Table 1), treatments combinations were defined by their concentrations of N (70, 120, 180, 250, and 290 mg L^–1^), P (20, 40, 60, 80, and 100 mg L^–1^), and K (60, 120, 200, 280, and 340 mg L^–1^) (Table 2) achieved by using different amounts of varies straight fertilisers (Table 3). The experimental unit (replicate) was one plant grown in an individual DWC unit. There were 15 different treatments, with at least five replicates per treatment. Plants were randomly assigned to each nutrient solution treatment by generating a random sequence of numbers from 1 to 100 arranged in ten columns and ten rows (matching DWC unit arrangement). For the first 3 weeks following transplant (vegetative growth), all plants received the same nutrient solution containing (mg L^–1^): 112.8 N-NO_3_, 7.2 N-NH_4_, 40 P, 180 K, 110 Ca, 45 Mg, and 60 S. Once switched to short-day conditions, plants received their respective treatment nutrient solutions for the remainder of the experiment. Rainwater was used to make the nutrient solutions. The major cation and anion compositions of the treatment nutrient solutions are detailed in Table 4. All treatments were formulated to have the same N-NH_4_/N-NO_3_ ratio (1:16). All plants received the same concentration of a commercial ethylenediaminetetraacetate (EDTA) and diethylenetriamine pentaacetate (DTPA) chelated micronutrient mix throughout both vegetative and flowering stages (Plant-Prod Chelated Micronutrient Mix; Master Plant-Prod Inc., Brampton, Ontario, Canada) containing (mg L^–1^): 2.1 Fe, 0.6 Mn, 0.12 Zn, 0.03 Cu, 0.39 B, and 0.018 Mo.

**TABLE 1:** Coded and un-coded factors for each treatment of the response surface analysis experiment investigating the effect of nitrogen (N), phosphorus (P), and potassium (K) rates on cannabis production in deep-water culture.

	Coded factors			Un-coded factors (mg L^–1^)		
		
Treatment	A	B	C	N^a^	P	K
1	–1	–1	–1	120	40	120
2	+1	-1	–1	250	40	120
3	–1	+1	–1	120	80	120
4	+1	+1	–1	250	80	120
5	–1	–1	+1	120	40	280
6	+1	–1	+1	250	40	280
7	–1	+1	+1	120	80	280
8	+1	+1	+1	250	80	280
9	–1.682	0	0	70	60	200
10	+1.682	0	0	290	60	200
11	0	–1.682	0	180	20	200
12	0	+1.682	0	180	100	200
13	0	0	–1.682	180	60	60
14	0	0	+1.682	180	60	340
15	0	0	0	180	60	200
16	0	0	0	180	60	200
17	0	0	0	180	60	200
18	0	0	0	180	60	200
19	0	0	0	180	60	200
20	0	0	0	180	60	200

*^*a*^N-NH_4_ + N-NO_3_.*

**TABLE 2:** Range and levels of the experimental factors according to three-factor central rotatable composite design.

	Range and levels				
	
Element	–1.68^b^	–1	0	1	1.68^b^
N^a^	70	120	180	250	290
P	20	40	60	80	100
K	60	120	200	280	340

*^*a*^N-NH_4_ + N-NO_3_.*

*^*b*^Radius adjustment factor for a three-factor design to make the design rotatable.*

**TABLE 3:** Amount of each straight fertiliser compound used to make treatment nutrient solutions.

	Fertiliser compound concentration (mg L^–1^)								
	
Treatment	Ca(NO_3_)_2_	KNO_3_	NH_4_NO_3_	KH_2_PO_4_	(NH_3_)H_2_PO_4_	K_2_SO_4_	KCl	MgSO_4_⋅7H_2_O	CaCl_2_⋅2H_2_O
1	650	180	–	180	–	–	–	450	–
2	1400	200	20	180	–	–	–	450	–
3	700	60	–	350	–	–	–	450	–
4	1550	60	–	350	–	–	–	450	–
5	600	200	–	180	–	–	300	450	50
6	1000	600	40	180	–	–	–	450	–
7	600	250	–	375	–	–	150	450	50
8	1100	500	30	350	–	–	–	450	–
9	150	350	–	250	20	–	–	450	300
10	1550	350	–	250	–	–	–	450	–
11	800	400	10	90	–	–	40	450	–
12	950	220	–	400	40	–	–	450	–
13	1100	–	–	220	–	–	–	450	–
14	450	700	40	260	–	–	–	450	150
15	800	400	–	200	50	–	–	450	–
16	800	400	–	200	50	–	–	450	–
17	800	400	–	200	50	–	–	450	–
18	800	400	–	200	50	–	–	450	–
19	800	400	–	200	50	–	–	450	–
20	800	400	–	200	50	–	–	450	–

**TABLE 4:** Composition of major anions and cations in the treatment nutrient solutions.

	Nutrient concentrations (mg L^–1^)						
	
Treatment	N	P	K	Ca	Mg	S^a^	Cl
1	120	40	120	130	45	180	5.0
2	250	40	120	260	45	180	5.0
3	120	80	120	130	45	180	5.0
4	250	80	120	260	45	180	5.0
5	120	40	280	130	45	180	190
6	250	40	280	190	45	180	5.0
7	120	80	280	130	45	180	120
8	250	80	280	190	45	180	5.0
9	70	60	200	130	45	180	190
10	290	60	200	260	45	180	5.0
11	180	20	200	130	45	180	20
12	180	100	200	160	45	180	5.0
13	180	60	60	190	45	180	5.0
14	180	60	340	130	45	180	95
15	180	60	200	130	45	180	5.0
16	180	60	200	130	45	180	5.0
17	180	60	200	130	45	180	5.0
18	180	60	200	130	45	180	5.0
19	180	60	200	130	45	180	5.0
20	180	60	200	130	45	180	5.0

*^*a*^Includes sulphur added by the sulphuric acid used to adjust pH of the nutrient solution.*

The initial pH of the nutrient solutions was adjusted to 5.6 with 1 M sulphuric acid or 1 M sodium hydroxide, as needed. DWC units were topped up with pH-adjusted (5.6) rainwater 3–4 days after each weekly nutrient solution replacement to replace water lost due to evapotranspiration. Nutrient solution pH and electrical conductivity (EC, mS cm^–1^) were measured using a hand-held metre (BLU2300E Combo Metre, Bluelab Corporation, New Zealand). EC and pH of treatment feed solution and of the final drained solution are listed in Table 5.

**TABLE 5:** Electrical conductivity (EC) and pH of feed and drain nutrient solutions.

Treatment	Feed EC (mS cm^–1^)	Drain EC (mS cm^–1^)	Feed pH	Drain pH
1	1.5	1.1	5.6	6.5
2	2.4	2.3	5.6	5.6
3	1.5	1.2	5.6	6.2
4	2.5	2.2	5.6	5.6
5	2.1	2.0	5.6	6.5
6	2.5	2.5	5.6	5.9
7	2.1	1.9	5.6	6.2
8	2.5	2.2	5.6	6.1
9	1.8	1.8	5.6	6.5
10	2.8	2.8	5.6	5.6
11	2.0	1.9	5.6	6.6
12	2.1	2.0	5.6	5.6
13	1.8	1.6	5.6	5.3
14	2.3	2.4	5.6	6.4
15	1.9	1.9	5.6	5.9
16	1.9	1.8	5.6	6.0
17	1.9	1.8	5.6	5.6
18	1.9	1.8	5.6	6.1
19	1.9	1.8	5.6	5.9
20	1.9	1.9	5.6	5.9

### Plant Measurements

#### Aboveground Growth

Plant height and spread of the first three plants in each treatment were measured during the fifth week of the flowering stage. Plant height (cm) was measured from the lid of the DWC unit to the top of the apical inflorescence, and plant spread (cm) was measured at the widest point on the plant and then perpendicular to this measurement. Growth index (GI) was then calculated using the formula [GI = (height × width_1_ × width_2_)/300] (Caplan et al., 2017a). Plants were destructively harvested during the eighth week of flowering. To assess aboveground (including inflorescence) fresh weight (FW), plants were cut at substrate level and individually weighed on a digital balance.

#### Root Weight

During harvest, roots from the first three replicates of each treatment were cut from around the outer surface of the mesh pot and air dried for several days and then oven-dried at 92°C for 72 h and weighed (EG2200-2NM, KERN & SOHN, Balingen, Germany) to obtain root dry weight (DW).

#### Inflorescence Yield

Inflorescence material was trimmed of leaf tissue, removed from the stem, and then weighed to obtain inflorescence fresh weight (g/plant). To determine inflorescence dry weight (i.e., yield), ∼25 g samples of fresh inflorescence material from the first three plants in each treatment were weighed, dried at 70°C for 72 h, and then re-weighed to obtain dry weight (DW). Yield was computed on a per-plant basis as the total inflorescence FW × (sample DW/sample FW). Cured “whole-bud” cannabis inflorescence sold commercially normally contains 10 to 15% water. Therefore, the marketable yield can be calculated from inflorescence DW by factoring in the appropriate water content.

#### Cannabinoid Content

Representative samples (∼50 g) of fresh inflorescence from three plants per treatment were dried at 18°C and 50% relative humidity until inflorescence material reached ∼10% moisture. Composite sub-samples (∼10 g) of air-dried inflorescence material from the first three replicates in each treatment were vacuum-sealed and sent to HEXO Corp’s in-house laboratory to determine cannabinoid concentration, including delta-9-tetrahydrocannabinol (Δ^9^-THC), tetrahydrocannabinolic acid (THCA), cannabidiol (CBD), cannabidiolic acid (CBDA), cannabigerol (CBG), cannabigerolic acid (CBGA), cannabichromene (CBC), cannabinol (CBN), and delta-8-tetrahydrocannabinol (Δ^8^-THC).

The cannabinoid analysis was conducted using ultra performance liquid chromatography (UPLC) separation. The composite sub-sample of dried cannabis was milled to a fine powder; from which1.0 g was extracted with an Acetontrile/H_2_O mixture with sonication and agitation for 20 min at ambient temperature. A 1.5 mL aliquot was diluted and filtered into a HPLC vial and analysed as per 7020006509EN (Layton and Aubin, 2019).

### Statistical Design and Analysis

RStudio software (RStudio Team, 2020) was used for data analysis. Normality and homoscedasticity of the data were assessed, and the data met these assumptions. The RStudio package “rsm” (Lenth, 2009) was used to analyse inflorescence yield and to generate three-dimensional and contour plots to represent the response surface. To improve the precision of yield estimates, the average yield of the five replicates in each treatment was used. Two sets of three surface and contour plots were created, each while holding one of the nutrient concentrations fixed at its centre point. These surface and contour plots, along with canonical analysis, were then used to determine the optimal rate of all three factors. Correlation analysis of yield and vegetative parameters was performed using the RStudio software package “ggplot2” (Wickham, 2016). To determine if there were differences in inflorescence cannabinoid content attributable to treatment, data from cannabinoid analysis was tested with a one-way ANOVA followed by Tukey’s HSD *post hoc* test.

#### Statistical Model

*yield* = μ + *n* + *n*^2^ + *p* + *p*^2^ + *k* + *k*^2^ + (*n* × *p*) + (*n* × *k*) + (*p* × *k*) + (n × p × k)

yield = dry inflorescence weight (g/plant)

μ = overall mean inflorescence weight (g/plant)

n = linear nitrogen component (fixed effect)

n^2^ = quadratic nitrogen component (fixed effect)

p = linear phosphorus component (fixed effect)

p^2^ = quadratic phosphorus component (fixed effect)

k = linear potassium component (fixed effect)

k^2^ = quadratic potassium component (fixed effect)

n × p = nitrogen and phosphorus interaction (fixed interaction effect)

n × k = nitrogen and potassium interaction (fixed interaction effect)

p × k = phosphorus and potassium interaction (fixed interaction effect)

n × p × k = nitrogen and phosphorus and potassium interaction (fixed interaction effect).

## Results

### Inflorescence Yield Response

**FIGURE 2:**
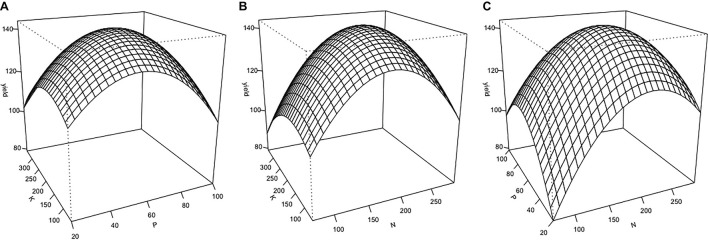
Three-dimensional response surfaces for inflorescence yield (g/plant) at a range of nutrient solution N, P, and K concentrations (mg L^–1^) of *Cannabis sativa* grown in deep water culture (*P* ≤ 0.05, *R*^2^ = 0.57). **(A)** Surface plot of K vs. P at N = 180 mg L^–1^. **(B)** Surface plot of K vs. N at P = 60 mg L^–1^. **(C)** Surface plot of P vs. N at K = 200 mg L^–1^.

**FIGURE 3:**
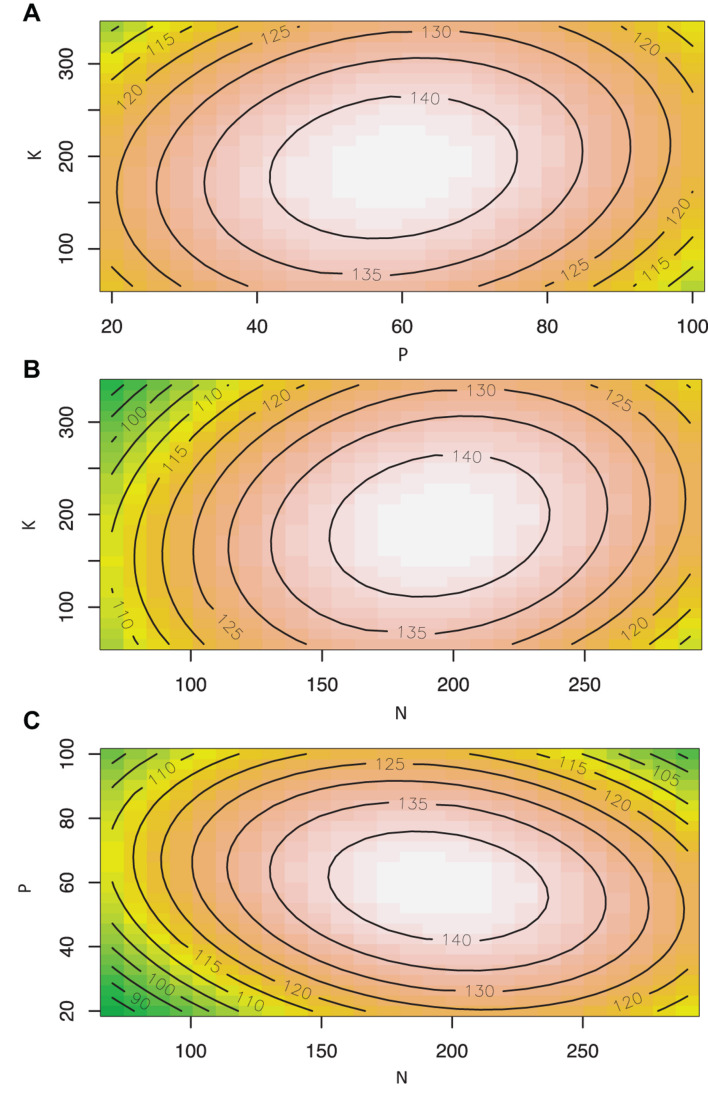
Contour plots showing the effect of nutrient solution N, P, and K concentrations (mg L^–1^) on inflorescence yield (g/plant) of *Cannabis sativa* grown in deep water culture (*P* ≤ 0.05, *R*^2^ = 0.57). **(A)** Contour plot of P vs. K at N = 180 mg L^–1^. **(B)** Contour plot of N vs. K at P = 60 mg L^–1^. **(C)** Contour plot of N vs. P at K = 200 mg L^–1^.

Cannabis inflorescence yield responded to increasing N and P supply but did not respond to K within the tested range (Figures 2, 3). Based on the surface response model, the estimated highest average yield of 144 g/plant would be achieved with N and P concentrations of 194 and 59 mg L^–1^, respectively. Visual analysis of contour graphs (with a 5 g resolution) show that yield responded to N best in the range of 160–230 mg L^–1^, and P in the range of 40–80 mg L^–1^ (Figure 2).

### Cannabinoid Content

There were no nutrient treatment effects on the inflorescence cannabinoid content. The average cannabinoid contents are listed in Table 6. In addition to those cannabinoids listed, the following were below the detection limits (i.e., <0.5 mg/g): CBC, CBD, CBDA, CBN, Δ^8^THC.

**TABLE 6:** Dry inflorescence average cannabinoid contents of *Cannabis sativa* grown in the deep-water culture system with different NPK concentrations in the solution.

Cannabinoid	Concentration in inflorescence (mg g^–1^)^b^
CBG	0.86 ± 0.01
CBGA	3.9 ± 0.08
THC	4.4 ± 0.09
THCA	161 ± 2.32
Total THC^a^	146 ± 2.06

*^*a*^Total THC = [THC] + 0.877[THCA].*

*^*b*^Mean ± SE (*n* = 60).*

### Relationships Between Inflorescence Yield and Vegetative Growth Attributes

**FIGURE 4:**
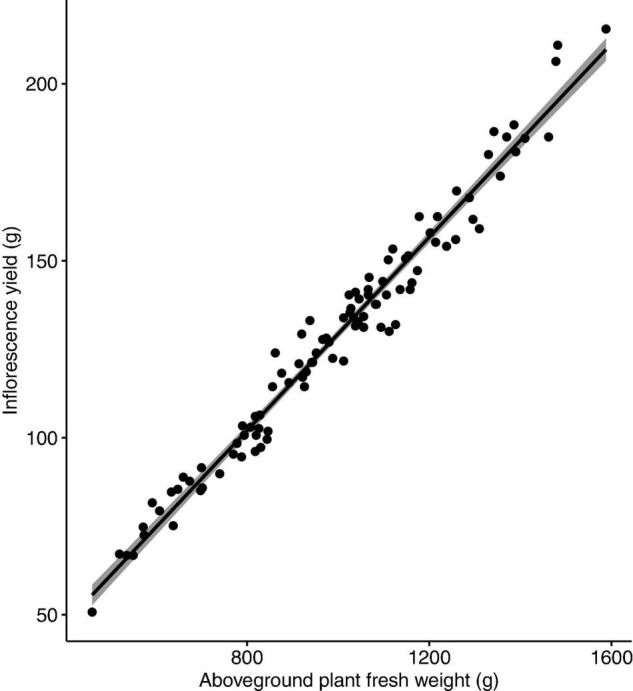
Correlation between inflorescence yield (g/plant) and aboveground plant fresh weight (g/plant) of *Cannabis sativa* (*r* = 0.98, *P* < 0.001). Shaded area represents 95% confidence interval.

**FIGURE 5:**
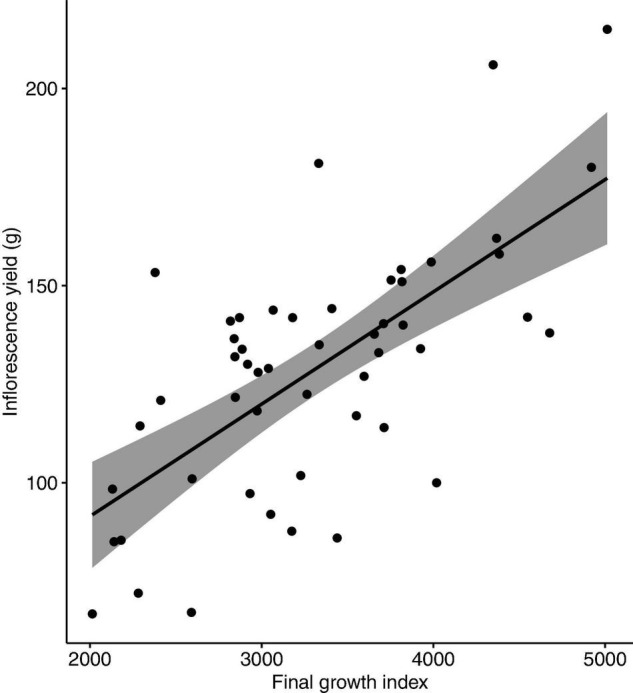
Correlation between inflorescence yield (g/plant) and plant growth index of *Cannabis sativa* (*r* = 0.67, *P* < 0.001). Shaded area represents 95% confidence interval.

**FIGURE 6:**
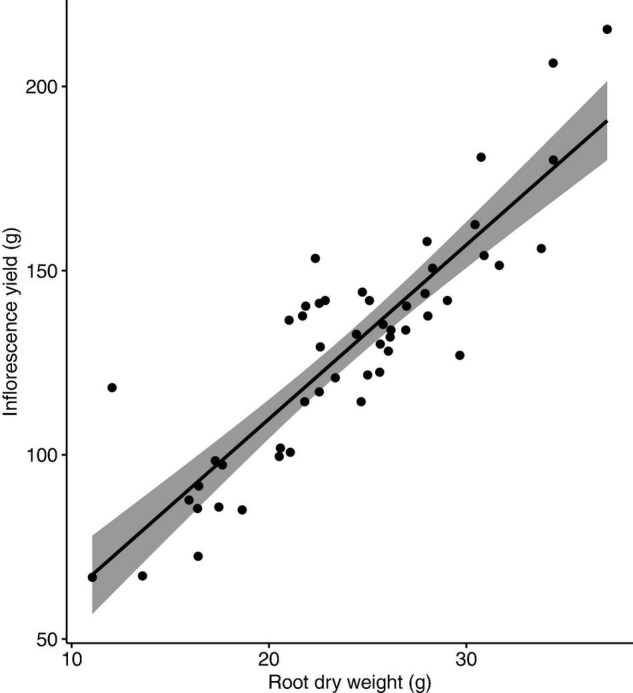
Correlation between inflorescence yield and root dry weight of *Cannabis sativa* (*r* = 0.9, *P* < 0.001). Shaded area represents 95% confidence interval.

No nutrient deficiency or toxicity symptoms were observed on any plants. Inflorescence yield was linearly and positively correlated with the measured vegetative growth attributes. Inflorescence yield had significant correlations with aboveground plant fresh weight (Figure 4), plant growth index (Figure 5), and root dry weight (Figure 6).

## Discussion

The goal of this study was to determine the optimal concentration of N, P, and K in the nutrient solution for the flowering stage of soilless cannabis production using RSM. The optimal concentrations of nutrient solution N and P was predicted to be approximately 194 mg L^–1^ N, and 59 mg L^–1^ P, respectively. Based on analysis of the response surface model, it was found N and P were the most important factors in predicting inflorescence yield. Inflorescence yield decreased markedly outside of the range of 160–230 mg L^–1^ N, and 40–80 mg L^–1^ P. These findings suggest that drug-type cannabis responds well to nitrogen and phosphorus during the flowering stage. Inflorescence yield did not respond to nutrient solution K concentration within the tested range, indicating the K currently supplied (300–400 mg L^–1^) by some commercial cultivators are likely too high.

Inflorescence yield had a strong positive correlation with a number of vegetative growth attributes. The strong correlation between inflorescence yield and plant growth index indicates that larger plant size can result in higher inflorescence yield. Nutrient supply, especially N, can determine cannabis plant size as N is an essential component of plant chlorophyll and ribulose-1,5-bisphosphate carboxylase-oxygenase (Rubisco). Low levels of N can reduce plant photosynthetic capacity and limit plant growth (Saloner and Bernstein, 2020). For flowering drug-type cannabis in soilless culture, supply of 30 and 80 mg L^–1^ N restricted whole plant and inflorescence growth, but plants performed optimally with supply of 160–320 mg L^–1^ N (Saloner and Bernstein, 2021). The optimal N supply (194 mg L^–1^) found in our study is within their range, despite the two studies using two different growing methods and plants with different genetic backgrounds. For drug-type cannabis during the flowering stage in an organic-based soilless production system, the optimal N supply was slightly higher (212–261 mg L^–1^; Caplan et al., 2017a) than the optimal level found in the present study. A possible explanation for the higher optimal N supply in the organic fertiliser study is that N from organic-based fertilisers may not always be readily available, as the release of N from organic fertilisers depends on the speed and extent of the mineralisation process (Hartz et al., 2010; Dion et al., 2020). Though it is unclear what source of organic nitrogen was used in their study, factoring in organic N availability of around 60% would put our findings in line with those by Caplan et al. (2017a). Along with aboveground growth, root growth also contributes to overall plant size. We found that inflorescence yield had a strong positive correlation with root dry weight, supporting our conclusion that larger plants produce higher yields. The context of where plants spend their energy is important. For industrial hemp, increasing N supply increased plant growth, but this growth was partitioned more towards stem material rather than valuable inflorescence material (Campiglia et al., 2017). Further investigations of cannabis response to nitrogen should consider product quality, and the distribution of biomass to various plant organs to maximise inflorescence growth and quality.

While modelling of cannabis inflorescence yield response to N, P, and K with surface analysis accounts for interaction between nutrients, the surface response model demonstrated that K, within the tested range of 60–340 mg L^–1^, had no effect on inflorescence yield. This lack of response may suggest that 60 mg L^–1^ K is not low enough to cause nutrient deficiency, and 340 mg L^–1^K is not high enough to cause toxicity. Moreover, cannabis responses to K may be genotype specific. Plants of one cannabis genotype Royal Medic supplied with 240 mg L^–1^ K had 25% reduced fresh shoot and root biomass by compared to those fed with 175 mg L^–1^, while plants of genotype Desert Queen had up to 40% increased shoot and root biomass (Saloner et al., 2019). Plant height, number of nodes on the main stem, and stem diameter of these two genotypes remained similar, so this difference in biomass was caused by one genotype becoming “bushier” than the other under high K supply. These differences in the response to K supply may be due to differences in plant tissue (e.g., main stem vs. side branch) sensitivity to K. Plant phenological stage (i.e., vegetative or flowering stage) may also be a factor in cannabis response to K supply. In a previous study of flowering aquaponic cannabis response to K, inflorescence yield increased when plants were provided with K up to 150 mg L^–1^ (Yep and Zheng, 2020). Genotype and plant phenological stage should be considered in future studies looking at cannabis response to nutrients, especially K.

Many commercial cannabis cultivation operations currently use fertiliser formulations that contain very high levels of P (more than 200 mg L^–1^ P in some cases). This practice is based on anecdotal evidence that P enhances inflorescence production. These concentrations are much higher than the optimal rate of 60 mg L^–1^ P found in our study, and at the higher range could cause reduction of both plant growth and inflorescence yield. In addition to reducing plant growth and yield, excessive supply of nutrients is a potential source of environmental pollution. Though, cannabis does appear to have the ability to store and mobilise certain amount of P when required. When provided with P higher than 30 mg L^–1^ in the vegetative stage, cannabis sequestered excess P in root tissue to prevent excess accumulation in the shoots (Shiponi and Bernstein, 2021). A greater understanding of what cannabis P requirements are, and whether there is any truth to the practice of supplying high concentrations of P, should be a priority for making cannabis production more sustainable. However, based on existing data it appears that the levels of P found in many cannabis specific commercial fertilisers are far higher than needed and could lead to negative environmental impacts.

While the cannabinoid concentrations in the floral tissues in our study did not respond to nutrient solution NPK concentrations, other studies indicate that plant mineral nutrition can affect production of secondary metabolites in cannabis (Caplan et al., 2017a; Saloner and Bernstein, 2021). There appears to be an inverse relationship between cannabis yield and potency, with cannabinoid concentrations decreasing as plant inflorescence yield increases. Inflorescence from plants supplied with 160 mg L^–1^N had approximately 30 and 20% lower concentrations of THCA and CBDA than plants supplied with 30 mg L^–1^N (Saloner and Bernstein, 2021). However, while nutrient stress and deficiency may enhance inflorescence cannabinoid content, this method is not ideal for optimising overall plant productivity as plants supplied with 160 mg L^–1^ N yielded twice that of those supplied with 30 mg L^–1^N. Cannabis grown in two organic growing media with different organic fertiliser rates (i.e., 57, 113, 170, 226, and 283 mg L^–1^N) had negative linear relationships between the concentrations of inflorescence THCA and CBGA and the fertiliser application rate for some of the treatment combinations (i.e., growing media and fertiliser rate) (Caplan et al., 2017a). However, for the most of the treatment combinations, fertiliser rates from 57 to 226 mg L^–1^N did not have any effects on THCA or CBGA concentrations; and the cannabinoid concentrations only dropped when the fertiliser rate increased to the highest level of 283 mg L^–1^N. The context of yield is again important when analysing differences in cannabinoid content as THCA concentrations dropped by ∼20% in the highest fertiliser rate, but inflorescence yield almost doubled vs. lowest fertiliser rate. As noted by Bernstein et al. (2019), an understanding of how nutrient supply influences cannabinoid concentrations would be an important step towards controlling and standardising the cannabinoid contents of medical cannabis. Cannabinoid concentrations are also important to recreational consumers, who rank THC and CBD concentrations among the most important factors when making purchasing decisions (Zhu et al., 2020). Given that cannabinoids are the compounds that make cannabis so uniquely valuable, more work needs to be done to investigate the effect of mineral nutrition on cannabis yield, and the relationship between yield and potency. Further work should also evaluate other compounds that are known to impact product quality.

The use of central-composite design allows experimenters to account for potential interactions between the different nutrients. This is important as nutrient interactions have been shown to affect plant nutrient uptake (Fageria, 2001; Rietra et al., 2017). A recent study found that high K supply decreased concentrations of Ca and Mg in cannabis leaf tissue, indicating antagonistic relationships between these positively charged ions (Saloner et al., 2019). An understanding of how combinations of nutrients at different concentrations affect crop growth, yield, and quality is important for the development of recommendations for the commercial cannabis industry. Had the same number of nutrients and nutrient levels as were included in this study been investigated with a traditional full-factorial design, many more nutrient solution treatment groups would have been required, compared to the number of treatment groups used in this study. The difference in number of treatment groups needed can be more pronounced as more factors (i.e., Ca, Mg) are included. Considering the high cost of cannabis and growing space in controlled environments, the response surface approach allowed us to complete this study where another experimental design may have been prohibitive.

No matter the experimental design used, an inherent problem in nutrient solution experiments is that nutrients cannot be added individually but must be added as a compound containing both anions and cations. Further, the ionic balance constraint requires the sum of the charges of cations and anions in solution to be equal (De Rijck and Schrevens, 1999b). The implication for formulating experimental treatment solutions is that it is practically impossible to change the level of one nutrient while keeping concentrations of all other nutrients the same. In this study, we focused on N, P, and K concentrations while attempting to keep all other nutrients at reasonable levels using commonly available horticultural fertiliser compounds. For example, potassium nitrate and calcium nitrate usually contribute to the bulk of nitrogen, potassium, and calcium in horticultural nutrient solutions (Resh, 2012). Formulating a high N, low K nutrient solution with these fertilisers results in higher levels of Ca than other nutrient solution treatments. Likewise, a low N, high K nutrient solution necessitates an additional source of K such as KCl, which would increase solution Cl concentration. Higher concentrations of nutrients such as Ca and Cl bring the potential for nutrient interactions which may affect experimental results. The lack of response to K in the range of 60–340 mg L^–1^ observed in our trial may be partially due to competition for uptake from Ca. Regarding experimental Cl levels, hydroponic cannabis has been shown to tolerate rates of 180 mg L^–1^ Cl with no impact on yield or potency (Yep et al., 2020a) so it is unlikely Cl levels limited plant growth in this trial. Though less than ideal in an experimental setting, there is no perfect solution for the problem of keeping all nutrient concentrations the same when formulating treatment solutions.

While this trial determined the theoretical optimum levels of N and P for the DWC growing method, these levels may not be definitive for all production methods or genotypes. Our trial was conducted in solution culture with weekly nutrient solution changes, and the EC and pH dynamics of our DWC units are likely different than other growing methods, meaning that plant nutrient availability and overall salinity of the nutrient solution would also likely be considerably different. Many commercial cannabis operations utilise substrate-based soilless cultivation systems, such as coir in containers, that may offer more nutrient and pH buffering capacity (Zheng, 2020). Having said that, our trial does represent or closely resemble some common soilless production practices, such as growing cannabis in rockwool, in the current cannabis production industry (Zheng, 2021). The treatments were applied only during the short-day period (i.e., flowering stage), and considering that plant nutrient requirement may vary at different development stages, the same experiment may also need to be conducted for the vegetative stage. Another limitation of our study was that we only used a single cannabis cultivar. Similar experiments should be performed on different cultivars, with disparate growth habits and cannabinoid compositions to investigate how individual cultivars may respond to NPK treatment levels. Additionally, this study only looked at inflorescence yield and cannabinoid composition and did not evaluate the impact of NPK on inflorescence terpene content or organoleptic properties.

Drug-type cannabis is still a relatively new crop in the legal setting, especially for large-scale commercial production, and many aspects of its cultivation are relatively unknown. We found that response surface methodology was a suitable experimental approach for investigation of cannabis responses to NPK, and that modelling of yield response to these nutrients aided us in achieving our experimental objective. Based on the results of this study, we recommend providing plants with a nutrient solution containing N and P at approximately 194 and 59 mg L^–1^, respectively, to achieve maximal inflorescence yield. Future studies should investigate the inflorescence yield and vegetative growth response of genetically diverse cultivars to macronutrients and include more quality parameters to ensure that plant yields do not compromise product quality. Improving our understanding of cannabis responses to mineral nutrients is an essential step towards the effective and sustainable cultivation of this high-value horticultural crop.

## Data Availability Statement

The original contributions presented in the study are included in the article/supplementary material, further inquiries can be directed to the corresponding author.

## Author Contributions

All authors designed the experiment and wrote the manuscript. LB conducted the trial and processed the data.

## Conflict of Interest

The authors declare that the research was conducted in the absence of any commercial or financial relationships that could be construed as a potential conflict of interest.

## Publisher’s Note

All claims expressed in this article are solely those of the authors and do not necessarily represent those of their affiliated organizations, or those of the publisher, the editors and the reviewers. Any product that may be evaluated in this article, or claim that may be made by its manufacturer, is not guaranteed or endorsed by the publisher.

## References

[B1] Beerling E. A. M. Blok C. van der Maas A. A. van Os E. A. (2014). Closing the water and nutrient cycles in soilless cultivation systems. *Acta Hortic.* 1034 49–55. 10.17660/ActaHortic.2014.1034.4

[B2] Bernstein N. Gorelick J. Zerahia R. Koch S. (2019). Impact of N, P, K, and humic acid supplementation on the chemical profile of medical cannabis (*Cannabis sativa* L.). *Front. Plant. Sci.* 10:736. 10.3389/fpls.2019.00736 31263470 PMC6589925

[B3] Campiglia E. Radicetti E. Mancinelli R. (2017). Plant density and nitrogen fertilization affect agronomic performance of industrial hemp (Cannabis sativa L.) in Mediterranean environment. *Ind. Crop. Prod.* 100 246–254. 10.1016/j.indcrop.2017.02.022

[B4] Caplan D. Dixon M. Zheng Y. (2017b). Optimal rate of organic fertilizer during the vegetative-stage for cannabis grown in two coir-based substrates. *HortScience* 52 1307–1312. 10.21273/hortsci11903-17

[B5] Caplan D. Dixon M. Zheng Y. (2017a). Optimal rate of organic fertilizer during the flowering stage for cannabis grown in two coir-based substrates. *HortScience* 52 1796–1803. 10.21273/hortsci12401-17

[B6] De Rijck G. Schrevens E. (1998). Multifactorial optimisation of the nutrient solution for hydroponically grown chicory plants. *Sci. Hortic.* 76 149–159. 10.1016/S0304-4238(98)00126-125

[B7] De Rijck G. Schrevens E. (1999a). Application of mixture theory for the optimisation of the composition of nutrient solutions for hydroponic cropping: practical use. *Acta Hortic.* 481 205–212. 10.17660/ActaHortic.1999.481.21

[B8] De Rijck G. Schrevens E. (1999b). Chemical feasibility region for nutrient solutions in hydroponic plant nutrition. *J. Plant Nutr.* 22 259–268. 10.1080/01904169909365624

[B9] Dion P.-P. Jeanne T. Thériault M. Hogue R. Pepin S. Dorais M. (2020). Nitrogen release from five organic fertilizers commonly used in greenhouse organic horticulture with contrasting effects on bacterial communities. *Can. J. Soil. Sci.* 100 120–135. 10.1139/cjss-2019-2056 33356898

[B10] Fageria V. D. (2001). Nutrient interactions in crop plants. *J. Plant Nutr.* 24 1269–1290. 10.1081/pln-100106981

[B11] Hartz T. K. Smith R. Gaskell M. (2010). Nitrogen availability from liquid organic fertilizers. *HortTechnology* 20 169–172. 10.21273/horttech.20.1.169

[B12] Layton C. Aubin A. J. (2019). *UPLC Separation for the Analysis of Cannabinoid Content in Cannabis Flowers and Extracts.* Available online at: https://www.waters.com/webassets/cms/library/docs/720006509en.pdf (accessed October 25, 2021).

[B13] Lenth R. V. (2009). Response-surface methods in R, using rsm. *J. Stat. Softw.* 32 1–17.

[B14] Myers R. H. Montgomery D. C. Anderson-Cook C. M. (2016). *Response Surface Methodology: Process and Product Optimization using Designed Experiments*, 4th Edn. Hoboken, NJ: Wiley.

[B15] Ontario Ministry of Agriculture Food and Rural Affairs (2019). *Nutrient Management- Greenhouse Nutrient Feedwater Regulation.* Guelph, ONT: OMAFRA.

[B16] Resh H. M. (2012). *Hydroponic Food Production: a Definitive Guidebook for the Advanced Home Gardener and the Commercial Hydroponic Grower*, 7th Edn. Boca Raton, FL: CRC Press, Taylor & Francis Group.

[B17] Rietra R. P. J. J. Heinen M. Dimkpa C. O. Bindraban P. S. (2017). Effects of nutrient antagonism and synergism on yield and fertilizer use efficiency. *Commun. Soil. Sci. Plant Anal.* 48 1895–1920. 10.1080/00103624.2017.1407429

[B18] RStudio Team (2020). *RStudio: Integrated Development Environment for R.* Boston, MA: RStudio.

[B19] Saloner A. Bernstein N. (2020). Response of medical cannabis (*Cannabis sativa* L.) to nitrogen supply under long photoperiod. *Front. Plant Sci.* 11:572293. 10.3389/fpls.2020.572293 33312185 PMC7704455

[B20] Saloner A. Bernstein N. (2021). Nitrogen supply affects cannabinoid and terpenoid profile in medical cannabis (*Cannabis sativa* L.). *Ind. Crop. Prod.* 167:113516. 10.1016/j.indcrop.2021.113516

[B21] Saloner A. Sacks M. M. Bernstein N. (2019). Response of medical cannabis (*Cannabis sativa* L.) genotypes to K supply under long photoperiod. *Front. Plant Sci.* 10:1369. 10.3389/fpls.2019.01369 31803198 PMC6876614

[B22] Schindler D. W. Carpenter S. R. Chapra S. C. Hecky R. E. Orihel D. M. (2016). Reducing phosphorus to curb lake eutrophication is a success. *Environ. Sci. Technol.* 50 8923–8929. 10.1021/acs.est.6b02204 27494041

[B23] Shiponi S. Bernstein N. (2021). Response of medical cannabis (*Cannabis sativa* L.) genotypes to P supply under long photoperiod: functional phenotyping and the ionome. *Ind. Crop. Prod.* 161:113154. 10.1016/j.indcrop.2020.113154

[B24] Wickham H. (2016). *ggplot2: Elegant Graphics for Data Analysis.* New York, NY: Springer-Verlag.

[B25] Yep B. Gale N. V. Zheng Y. (2020b). Comparing hydroponic and aquaponic rootzones on the growth of two drug-type *Cannabis sativa* L. cultivars during the flowering stage. *Ind. Crop. Prod.* 157:112881. 10.1016/j.indcrop.2020.112881

[B26] Yep B. Gale N. V. Zheng Y. (2020a). Aquaponic and hydroponic solutions modulate NaCl-induced stress in drug-type *Cannabis sativa* L. *Front. Plant Sci.* 11:1169. 10.3389/fpls.2020.01169 32849724 PMC7424260

[B27] Yep B. Zheng Y. (2020). Potassium and micronutrients fertilizer addition in aquaponic solution for drug-type *Cannabis sativa* L. cultivation. *Can. J. Plant Sci.* 101 341–352. 10.1139/cjps-2020-2107 33356898

[B28] Zheng Y. (2018). Current nutrient management practices and technologies used in North American greenhouse and nursery industries. *Acta Hortic.* 1227 435–442. 10.17660/ActaHortic.2018.1227.54

[B29] Zheng Y. (2020). Integrated rootzone management for successful soilless culture. *Acta Hortic.* 1273 1–8. 10.17660/ActaHortic.2020.1273.1

[B30] Zheng Y. (2021). Soilless production of drug-type *Cannabis sativa*. *Acta Hortic.* 1305 376–382. 10.17660/ActaHortic.2021.1305.49

[B31] Zhu B. Guo H. Cao Y. An R. Shi Y. (2020). Perceived importance of factors in cannabis purchase decisions: a best-worst scaling experiment. *Int. J. Drug Policy* 91:102793. 10.1016/j.drugpo.2020.102793 32482489 PMC7704653

